# Improving medication safety on critical care using an anonymous electronic medication incident reporting system

**DOI:** 10.1186/cc9906

**Published:** 2011-03-11

**Authors:** H Dillon, M Rosbergen, R Wyatt, J Nortje

**Affiliations:** 1 Norfolk & Norwich University Hospital, Norwich, UK

## Introduction

To improve medication safety on the Critical Care Complex (CCC), Norfolk & Norwich University Hospital, an anonymous electronic reporting system was introduced. Reports captured populate a local database of incidents, which identifies themes. Medication incidents are common; studies reveal up to 10.5 incidents per 100 bed-days [1]. Under-reporting of incidents in the CCC was highlighted in a paper-based 2-week reporting project. The electronic reporting system expands this work, introducing a sustainable, integrated reporting system, addressing some of the reporting barriers.

## Methods

A staff survey identified barriers to incident reporting such as access to forms, time taken to complete reports and fear of disciplinary action. An anonymous medication incident system was developed and implemented in the bedside clinical information system, Metavision^®^. One-to-one education sessions highlighted the system and a survey informed optimal form design. Incidents reported were entered into a database and categorised by time, error types and themes. The database allowed identification of processes needing improvement. Subsequently, targeted changes to the systems surrounding medications were introduced to reduce specific incident types.

## Results

**Figure 1:**
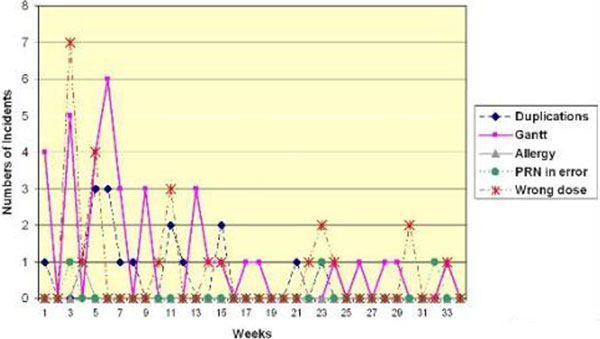
**Effect of system changes**.

Over 34 weeks, 194 medication incidents were reported. The most common types of incidents were infusion documentation (Gantt), wrong dose, duplication, wrong rate and wrong frequency errors. System changes in response to these errors have reduced their incidence (Figure 1).

## Conclusions

Incident reporting has improved significantly from a baseline of 19 reports in 2 years. The new reporting system has enabled targeted changes, eliminating some of the most common errors, improving medication safety. Fluctuating numbers of reports may still indicate under-reporting. Themes remain that have yet to be addressed.
